# Endosymbiont-derived metabolites are essential for tick host reproductive fitness

**DOI:** 10.1128/msphere.00693-23

**Published:** 2024-07-02

**Authors:** Balasubramanian Cibichakravarthy, Neta Shaked, Einat Kapri, Yuval Gottlieb

**Affiliations:** 1 The Robert H. Smith Faculty of Agriculture, Food and Environment, Koret School of Veterinary Medicine, The Hebrew University of Jerusalem, Rehovot, Israel; University of Michigan, Ann Arbor, Michigan, USA

**Keywords:** symbiosis, reproductive fitness, ticks, metabolism

## Abstract

Ticks, like other obligatory blood-feeding arthropods, rely on endosymbiotic bacteria to supplement their diet with B vitamins lacking in blood. It has been suggested that additional metabolites such as L-proline may be involved in this nutritional symbiosis, but this has yet to be tested. Here, we studied the metabolite-based interaction between the brown dog tick *Rhipicephalus sanguineus* (Acari: Ixodidae) and its *Coxiella-*like endosymbionts (CLE). We measured amino acid titers and tested the effect of B vitamins and L-proline supplementation on the fitness of CLE-suppressed female ticks, displaying low titers of CLE. We found higher titers of L-proline in the symbiont-hosting organs of unfed ticks and in engorged blood-fed whole ticks. Supplementation of B vitamins increased the hatching rate of CLE-suppressed ticks; this effect appears to be stronger when L-proline is added. Our results indicate that L-proline is produced by CLE, and we suggest that CLE is essential in states of high metabolic demand that affects tick reproductive fitness, such as oogenesis and embryonic development. These findings demonstrate the broader effect of nutritional symbionts on their hosts and may potentially contribute to the control of ticks and tick-borne diseases.

**IMPORTANCE:**

*Coxiella*-like endosymbionts (CLE) are essential to the brown dog tick *Rhipicephalus sanguineus* for feeding and reproduction. This symbiosis is based on the supplementation of B vitamins lacking in the blood diet. The involvement of additional metabolites has been suggested, but no experimental evidence is available as yet to confirm a metabolic interaction. Here, we show that B vitamins and L-proline, both of which contribute to tick reproductive fitness, are produced by CLE. These findings demonstrate the importance of symbiont-derived metabolites for the host’s persistence and shed light on the complex bacteria-host metabolic interaction, which can be channeled to manipulate and control tick populations.

## INTRODUCTION

Many animals live in association with bacteria that shape various aspects of their biology including nutritional status, coping with biotic and abiotic stress, and behavior. In arthropods, obligate associations with bacterial symbionts are relevant to a range of nutritional niches (1). Many associations between arthropods and nutritional symbionts promote supplementation of essential nutrients such as amino acids and B vitamins, enabling hosts to survive on nutrient-deficient diets such as plant sap or vertebrate blood. Aphids, for instance, harbor *Buchnera*, an intracellular bacterium that supplies essential amino acids lacking in phloem (2). Similarly, obligatory blood-feeding arthropods, including tsetse flies and bed bugs, form close associations with the endosymbiotic bacteria *Wigglesworthia* and *Wolbachia*, respectively (3, 4). These associations have been shown to be essential for the fitness and reproduction of the host, principally by providing B vitamins absent from the blood diet (5). In tsetse flies, the symbiont is also involved in the production of the amino acid L-proline, which is essential for reproduction (3).

The brown dog tick *Rhipicephalus sanguineus* (Acari: Ixodidae) harbors obligate *Coxiella-*like endosymbionts (CLE). Testing symbiont-host interactions in hard ticks (Ixodidae) can be challenging due to the lack of an artificial feeding system that can support a full developmental cycle for most ticks, thus requiring animals as feed and compromising on effective means to administer treatments of interest. Nevertheless, we previously demonstrated that the suppression of CLE by antibiotic treatment results in reduced host fitness and fecundity (6). CLE, which are maternally transmitted, are thought to provide nutrients lacking in the blood diet of their hosts, as do other nutritional mutualists of blood feeders (7). In *R. sanguineus*, they are localized in the Malpighian tubules (MT) and ovaries (OV) (8), but in other tick species, they may also be found in the salivary glands (SG) and midgut (MG) (9). CLE have the genetic capacity to synthesize several B vitamins and cofactors (7, 10, 11) and can also synthesize and transport L-proline (12). While *in silico* steady-state flux distribution using flux balance analysis of CLE genomes predicts zero sum of flux for B vitamins, it did reveal excretion of L-proline (12). Confirming the genomic postulations, the proteomics analysis of CLE identified the production of the proteins responsible for B vitamin and L-proline synthesis, such as BioB, BioF, FolE, BioD, RibE, PdxA, and ornithine cyclodeaminase, which converts L-ornithine to L-proline (13). Based on these indirect observations, we presume that B vitamins and L-proline synthesized by CLE are essential for the nutritionally based symbiosis between CLE and the brown dog tick. To test this hypothesis, we measured L-proline titers in symbiotic female ticks naturally harboring CLE and in CLE-suppressed females showing low titers of CLE following antibiotic treatment. Measurements were performed on unfed and fully fed ticks, and we tested the effect of supplementing B vitamins and L-proline on the fitness of CLE-suppressed female brown dog ticks. We found higher L-proline titers and lower L-ornithine titers in CLE-hosting organs (Malpighian tubules and ovaries) of symbiotic ticks than in those of CLE-suppressed ticks, suggesting that CLE converts L-ornithine to L-proline. Additionally, we found higher titers of L-proline in symbiotic than in CLE-suppressed engorged ticks and no differences in L-proline titers between symbiotic and CLE-suppressed unfed ticks. We also demonstrate that supplementing B vitamins and L-proline to engorged CLE-suppressed ticks improves the hatching rate of their eggs. Altogether, we suggest that CLE metabolite supplementation is required when ticks are subject to high metabolic demands during, for example, feeding and development.

## MATERIALS AND METHODS

### Tick maintenance

Fully engorged *Rhipicephalus sanguineus sensu lato* nymphs (*n* = 1,161) were purchased from the Oklahoma State University Tick Rearing Facility (Stillwater, OK, USA) after approval from the Israel Ministry of Agriculture (Permit #19/2019). At the facility, tick colonies were routinely maintained on sheep, *Ovis aries* (L. Coburn, personal communication). Live engorged nymphs were shipped to our laboratory in plastic containers packed with moist paper toweling to maintain a humid environment. The individual nymphs were weighed and then held individually in cap-perforated tubes in a dark climate chamber (25°C ± 1°C, 85% ± 5% RH) until use. We conducted two separate experiments to obtain sufficient individual tick replicates. The details of each experiment are shown in Table S1.

### Suppression of *Coxiella-*like endosymbionts

**Fig 1:**
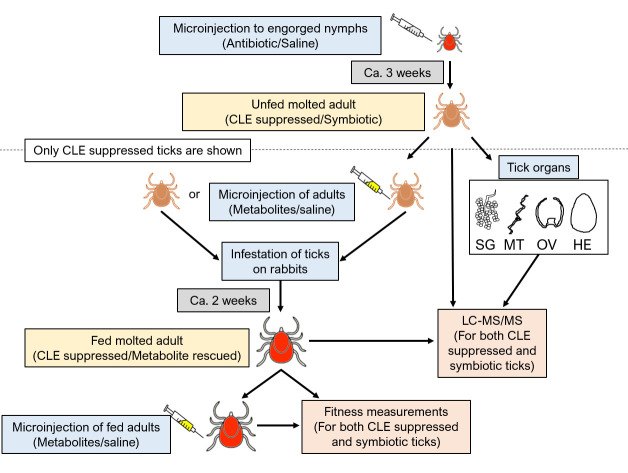
Experimental design. Top: retrieving symbiotic (saline treated, natural CLE titer) and antibiotic-treated (low CLE titer) ticks. Lower left: rescue treatments with metabolites (B vitamins and L-proline) or saline (control) to adult ticks before and after feeding (only antibiotic-treated ticks are shown). Lower right: retrieving unfed whole adult ticks and their organs after antibiotic/saline treatments to nymphs, and fed adult ticks after metabolite/saline treatments for amino acid analysis. SG-salivary glands; MT- Malpighian tubules; OV- ovaries; HE- Hemolymph. The number of ticks used for each experimental step is shown in the “Number” tab in Table S1.

Antibiotic suppression of CLE was performed at the nymphal stage, while the experiments and tests were performed on the subsequently emerging adults as previously described (6). This method avoids direct effects of antibiotics on the adult stage but does not exclude indirect effects established during nymphal molting. Engorged nymphs were treated with ofloxacin antibiotics (Sigma-Aldrich, Israel; Exp. 1, *n* = 400; Exp. 2, *n* = 478) or with saline (Exp. 1, *n* = 122; Exp. 2, *n* = 162) immediately after weighing to prevent cuticle hardening. Since sexual dimorphism of *R. sanguineus* is detectable only in adults, the exact number of female ticks was known only at a later stage. For treatment, 40–50 nymphs were gently transferred to an elevated inclined platform pre-taped with an adhesive band. They were then injected with an individually adjusted volume of ofloxacin to deliver a dose of 50 ng antibiotic per mg of body weight; controls received an equivalent volume of phosphate-buffered saline (PBS) as previously described (6). Treated nymphs were placed individually in cap-perforated 1.5 mL tubes and maintained in a dark climate chamber (25°C ± 1°C, 85% ± 5% RH) to complete their post-feeding development. During the following weeks, we daily counted and sexed adults that had newly matured from the treated nymphs until all had completed their development. The adults from each treatment group were weighed, and their scutal index was determined as previously described (6) (Fig. 1; Table S1).

### Validation of CLE suppression

The antibiotic treatment method was validated by measuring the CLE titer of five randomly chosen female ticks from each treatment group (antibiotic and saline) (Table S1). Genomic DNA was extracted using the DNeasy Blood and Tissue Kit (Qiagen, Hilden, Germany) according to the manufacturer’s instructions. In addition, nine engorged adult female ticks chosen randomly from the CLE-suppressed and control groups and used for metabolite extraction were also tested for CLE titer (Table S2, see below) . CLE-specific 16S rRNA and tick 18S rRNA gene copies were quantified using SYBR green-based qPCR on a SteponePlus machine (Applied Biosystems, CA, USA). The samples were run in triplicate, and the ratio between the two gene amplification titers was analyzed as previously described (6, 8, 14).

### Dissection of tick organs

Four to five unfed adult female ticks were chosen randomly from each treatment and surface sterilized in 70% ethanol for 30 seconds followed by three 1-min washes with ddH_2_O. They were then dissected to retrieve the salivary glands (SG), hemolymph (HE), Malpighian tubules (MT), and ovaries (OV) (Fig. 1; Table S3). The dissection was performed under a stereo microscope according to published protocol (15). The dissected organs from four ticks were pooled in a test tube with 50 µL of PBS. This procedure was repeated in four replicates for a total of 16 organ pools.

### Metabolite extraction and quantification

Ticks and organ samples were first lyophilized and then frozen in liquid nitrogen and held at −80°C until extraction. Before extraction, glass beads were added, two 2 mm beads for whole ticks and ten 1 mm beads for organ pools. To extract metabolites, 250 µL of methanol (MtOH) and a standard of 0.01 µg/mL proline-D_7_ (Cambridge Isotope Laboratories, USA) were added to each sample. The samples were then crushed in a FastPrep 24 homogenizer (MPbio, USA) using a program of 2× (4 m/second, 20 seconds), incubated for 3 min in an ice bath, and centrifuged at 500 rcf for 10 min at 4°C. The upper liquid phase containing the metabolites was collected and placed in an ice bath. The initial test tubes containing the pellet underwent a repeated process of crushing and methanolic extraction; the samples were then rotated in an Intelli-mixer ELM-RM-2M (ELMI, Latvia) at 4°C for 30 min. The test tubes were centrifuged once more at 500 rcf for 10 min at 4°C. The upper liquid was added to the liquid phase that was collected previously to obtain a total volume of 500 µL of extract containing metabolites. All samples were kept frozen at −80°C until sent for analysis by LC-MS/MS (liquid chromatography with tandem mass spectrometry). The remaining pellet was saved and used for further extraction of nucleic acids and proteins using an RNA/DNA/Protein Purification Plus Micro Kit (Norgen Biotek Corp, Thorold, ON, Canada) according to the manufacturer’s protocol. Finally, proteins were dissolved in 30 µL of elution buffer containing protease inhibitors (Promega, USA) according to the manufacturer’s protocol and measured using a BCA colorimetric kit (Thermo Fisher Scientific, USA).

Proteins and metabolites were analyzed by LC-MS/MS in the Targeted Metabolomics Unit, Weizmann Institute of Science, Israel

#### Protein hydrolysis

A volume of 200 µL of 6 N HCl was added to each protein suspension (20 µL), which was then vortexed and transferred to 12 mL Kimax tubes. The tubes were bubbled for 2 min with Ar-gas using tips for protein gel electrophoresis and then tightly sealed. The samples were heated in a dry heating block (Thermo Reactivap) from 25°C to 160°C for 1 h and then maintained at 160°C for 1 h, after which they were allowed to cool and were dried in an N_2_ flow. The resulting hydrolysates were re-suspended in 1 mL of 0.1% formic acid and taken for derivatization and LC-MS amino acid analysis.

#### Derivatization

Derivatization was performed with an AccQTag Ultra Kit (Waters). As per the manufacturer’s instructions, AccQTag reagent (20 µL) and internal standards (0.01 µg/mL proline-D_7_ and 60 µM arginine-^13^C_6_, 1 µL of each) in 0.15 M borate buffer, pH 8.8 (68 µL), were added to the 10 µL sample. The mixture was stirred at 55°C for 10 min. Prior to LC-MS/MS analysis, the samples were filtered through 0.2 µm PVDF filters (Millex GV) to remove insoluble materials.

#### LC-MS/MS analysis

Analysis was carried out using an LC-MS/MS instrument consisting of an Acquity I-class UPLC system (Waters) and a Xevo TQ-S triple quadrupole mass spectrometer (Waters) equipped with an electrospray ion source and operated in positive ion mode. MassLynx and TargetLynx software (version 4.1, Waters) were employed to acquire and analyze data. Chromatographic separation was performed on a 100 × 2.1 mm i.d. 1.8 µm UPLC HSS T3 column equipped with 50 × 2.1 mm i.d., 1.8 µm UPLC HSS T3 pre-column (both Waters Acquity) with 0.1% formic acid as mobile phase A and 0.1% formic acid in acetonitrile as B at a flow rate of 0.6 mL/min and column temperature 45°C. The A gradient was as follows: the column was held for 0.5 min at 4% B, then underwent a linear increase to 10% B in 2 min, then to 28% B in 2.5 min, and to 95% B in 0.1 min. Samples kept at ambient temperature (23°C) were automatically injected with a volume of 1 µL of the sample. For mass spectrometry, argon was used as the collision gas at a flow rate of 0.10 mL/min. The capillary voltage was set at 3.00 kV, cone voltage at 25 V, source offset at 30 V, source temperature at 150°C, desolvation temperature at 650°C, desolvation gas flow at 800 L/h, and cone gas flow at 150 L/h. Analytes were detected using corresponding selected reaction monitoring and retention times. Concentrations were calculated based on standard curves using TargetLynx (Waters).

### Obtaining engorged adult female ticks

To obtain engorged adult female ticks for metabolite quantification and to track egg production and hatching rates in later experiments, we mated and fed ticks on rabbits in a procedure following Almazán et al. (16), with slight modifications (Fig. 1; Table S1). New Zealand rabbits (*Oryctolagus cuniculus*) were purchased from Envigo Israel. Following a 1-week accommodation period, infestation with ticks was conducted under sedation of the rabbits by intramuscular injection of a mixture of Midazolam (Rafa Laboratories, Jerusalem, Israel) and Butorphanol (Butomidor, Richter Pharma, Austria) (0.5 mg/kg of body weight). For each treatment group, 15 female and 5 male unfed adult ticks were enclosed in a 6 × 9 cm capsule made of 5 mm low-density EVA foam (Cat. No. EVA-PE451 kg, Cosplay, Belgium) with a mesh cover, which was then attached to the rabbit host.

### Supplementation of B vitamins and L-proline to CLE-suppressed ticks

To evaluate the importance of B vitamins and L-proline, a rescue treatment was designed for CLE-suppressed ticks. We injected either B vitamins or B vitamins + L-proline, as well as a saline control, to CLE-suppressed and symbiotic adult females in two different feeding states: before feeding and infesting the rabbit host, and after feeding and repletion from the rabbit host. In order to avoid CLE transmission *via* co-feeding, control symbiotic ticks were placed on a separate rabbit (Fig. 1; Table S1).

#### B vitamins and L-proline solutions

A 10× aqueous cocktail of B vitamins was prepared according to previous supplementation experiments performed in artificial blood diet systems (4, 17, 18). The 10× stock solution contained thiamine (100 µg/mL), riboflavin (20 µg/mL), nicotinic acid (100 µg/mL), pantothenic acid (100 µg/mL), pyridoxine (100 µg/mL), D-biotin (1 µg/mL), folic acid (30 µg/mL), cobalamin (1 µg/mL), choline chloride (185 µg/mL), and meso-inositol (118 µg/mL). NaOH (1 M) was added to produce a transparent solution in spite of insolubility. The L-proline solution (300 mg/L) was prepared according to its concentration in tick cell line culture media (19). For tick treatment, we injected 1× of the B vitamin solution (B) or of the L-proline solution mixed with B vitamin solution (BP). Symbiotic tick controls were injected with saline.

#### Rescue treatment of unfed adult female ticks

Intact adult females that matured from the antibiotic-treated nymphs were placed on the inclined platform for the rescue treatment, which was performed by microinjecting a metabolite solution (either B vitamins or B vitamins + L-proline). The injection site and the tip of the needle were surface sterilized with 70% ethanol using a fine brush. Either the metabolite solution (B or BP) or the saline solution was injected into the adult ticks by inserting the needle tip adjacent to the coxa of the left foreleg while pointing away from the body center. A single injection volume of 90 nL was discharged at a rate of 5 nL/second, and the needle was left in place for an additional 30 seconds before retraction to allow the solution to disperse in the hemocoel. The needle was then retracted and allowed to discharge the solution to confirm that the tip was not blocked by tissues or hemolymph. Fifteen injected females and five untreated males from each treatment group were confined individually in capsules on a rabbit. Tick attachment and feeding progress were monitored daily for 2 weeks; engorged replete females were collected and counted for an additional week. After tick recovery was confirmed by observing active movement, each female was weighed to the nearest 0.01 mg, placed in a 2 mL cap-perforated tube, and maintained in a dark climate chamber (25°C ± 1°C, 85% ± 5% RH) for post-feeding development and oviposition.

#### Rescue treatment of engorged adult female ticks

The rescue treatment was applied to mated, fully fed replete females that received antibiotics as nymphs. Fifteen females were used for each treatment group (B; BP; and saline). Following repletion from the rabbit, ticks were weighed and injected with B, BP, or saline solution. After recovery, each individual female was weighed, placed in a cap-perforated tube, and maintained in a dark climate chamber for post-feeding development and oviposition.

#### Control treatment

Control (symbiotic) ticks were confined at a ratio of 3:1 untreated females to males in capsules placed on the rabbit. Tick attachment and feeding progress were monitored daily for 2 weeks, and engorged females leaving the host were collected and counted every 24 h. Replete females were weighed, placed individually in cap-perforated tubes, and maintained in the dark climate chamber for post-feeding development and oviposition.

### Data analysis

#### Assessment of CLE reduction following antibiotic treatment

The relative density of CLE was measured using qPCR on antibiotic-treated and saline-treated ticks (*n* = 5 biological replicates in each group). DNA isolation, target-specific primers, and qPCR conditions were as previously described (6). The results were analyzed using the Student’s *t*-test.

#### Metabolite comparison between symbiotic and CLE-suppressed ticks

The obtained quantities of L-proline- and L-ornithine-free and bound amino acids were first normalized to the total amino acids in each tick. A multivariate dependent variable analysis was performed to test the differences between symbiotic and aposymbiotic tick organs. Among the organs themselves, a Tukey-Kramer test and a *t*-test analysis were conducted (significance level *α* < 0.05). For entire symbiotic vs entire CLE-suppressed ticks and fed vs unfed ticks, an analysis of one dependent variable was performed between two treatment groups. Python 3.11 (Python.org) was used for all tests.

#### Rescue treatment analyses

The normal distribution of the data was tested using the Shapiro-Wilk test. Data that were normally distributed were analyzed using ANOVA, and a *t*-test was used for pairwise comparisons. For data that were not normally distributed, we used the non-parametric Kruskal-Wallis test followed by the Wilcoxon test for pairwise comparisons.

The specific test and replicates for each analysis are indicated in the corresponding figure legends. All tests were conducted using JMP Statistical Software, version 16.0 (JMP Statistical Discovery LLC, Cary, NC, USA) (*P* < 0.05). All the data were plotted using GraphPad Prism 8 software (https://www.graphpad.com/scientific-software/prism/). Unless specifically stated, sample size *n* means biological replication, and each point on the graphs represents a biological replicate.

## RESULTS

### Effect of antibiotic treatment on molting duration and suppression of CLE titers in emerged adults

Antibiotic and saline treatments were performed on 878 and 284 engorged nymphs, respectively. Nineteen days post-injection, 778 of the antibiotic-injected nymphs and 242 of the saline-injected nymphs had molted into adults. A considerable number of nymphs (*n* = 142) failed to molt and died, possibly due to physical injury during the microinjection or fungal contaminants. Ticks treated with saline molted significantly earlier than those treated with antibiotics, and females had a shorter development time than males, as we previously showed (Fig. S1) (6). After molting, the CLE titer of five random adult female ticks from each treatment group was measured. qPCR results demonstrated a significant reduction in CLE titer in the antibiotic-treated ticks compared with controls, as expected (Fig. S2a) (6). Titers of CLE were also tested in the engorged adult females used for metabolite extractions (Fig. S2b). While CLE titers in fed ticks varied within both the antibiotic-treated and symbiotic groups, the titer was significantly lower in the antibiotic-treated ticks.

### Effect of antibiotic treatment on physiological parameters and amino acid titer

**Fig 2:**
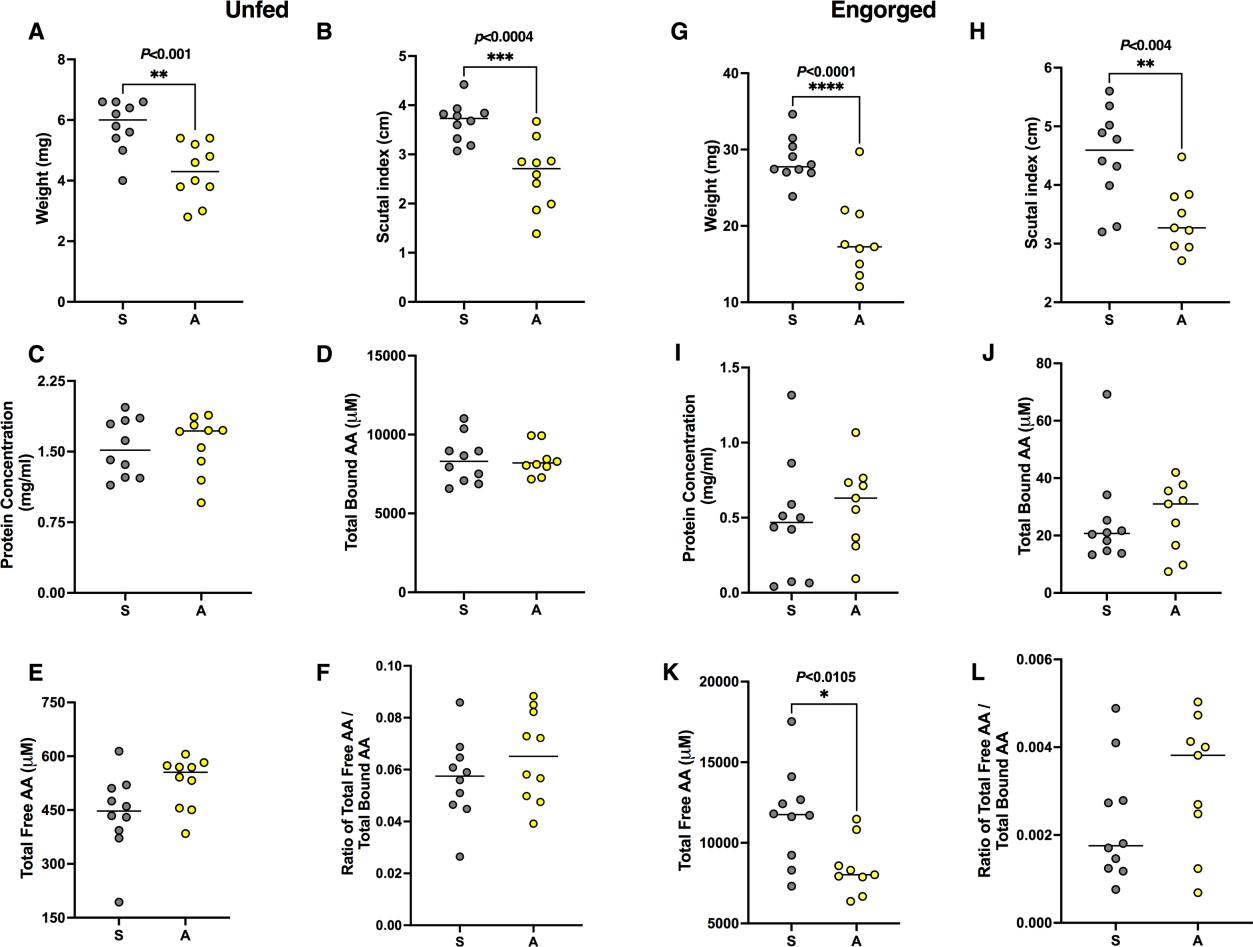
Physiological parameters and amino acid titers in saline- (S, gray) and antibiotic- (A, yellow) treated ticks (corresponding to symbiotic and suppressed CLE ticks). (**A–F**) Saline- and antibiotic-treated unfed ticks; (**G–L**) saline- and antibiotic-treated fed ticks. Shown are weight (**A and G**), scutal index (**B and H**), protein concentration (**C and I**), total bound amino acids (**D and J**), total free amino acids (**E and K**), and ratio between total free and bound amino acids (**F and L**) of individual ticks. Weight (**A**) and scutal index (**B**) of unfed saline-treated tick show significant differences (**P < 0.001, *t* = 3.82 and ***P < 0.0004, *t* = 4.27, respectively). Similarly, weight (**G**), scutal index (**H**), and total bound amino acid (**I**) of engorged treated ticks show significant differences (****P < 0.0001, *t* = 5.204; **P < 0.004, *t* = 3.320; and *P < 0.01, *t* = 2.872, respectively). *n* = 10 in each parameter tested for unfed ticks and engorged saline-treated ticks, and *n* = 9 in each parameter tested for engorged antibiotic-treated ticks; horizontal line depicts the median.

Physiological parameters such as scutal index, weight, and total protein content were evaluated in the same treated and control ticks used for measuring metabolites (Tables S2 and S3). The molted adult ticks that received the antibiotic treatment showed a lower scutal index and weight, but their protein content and total free and bound amino acids, as well as the ratio between the two, did not differ from those of molted adult ticks from the saline treatment (Fig. 2A through F). The engorged ticks used for metabolite measurements also differed significantly from controls in the scutal index and body weight, as well as in total free amino acids (Fig. 2G, H, and K). No differences in total protein, bound amino acids, and the ratio between total free and bound amino acids were found between engorged ticks that received antibiotics and controls (Fig. 2I, J, and L).

### Effect of antibiotic treatment on L-proline titer in engorged ticks

**Fig 3:**
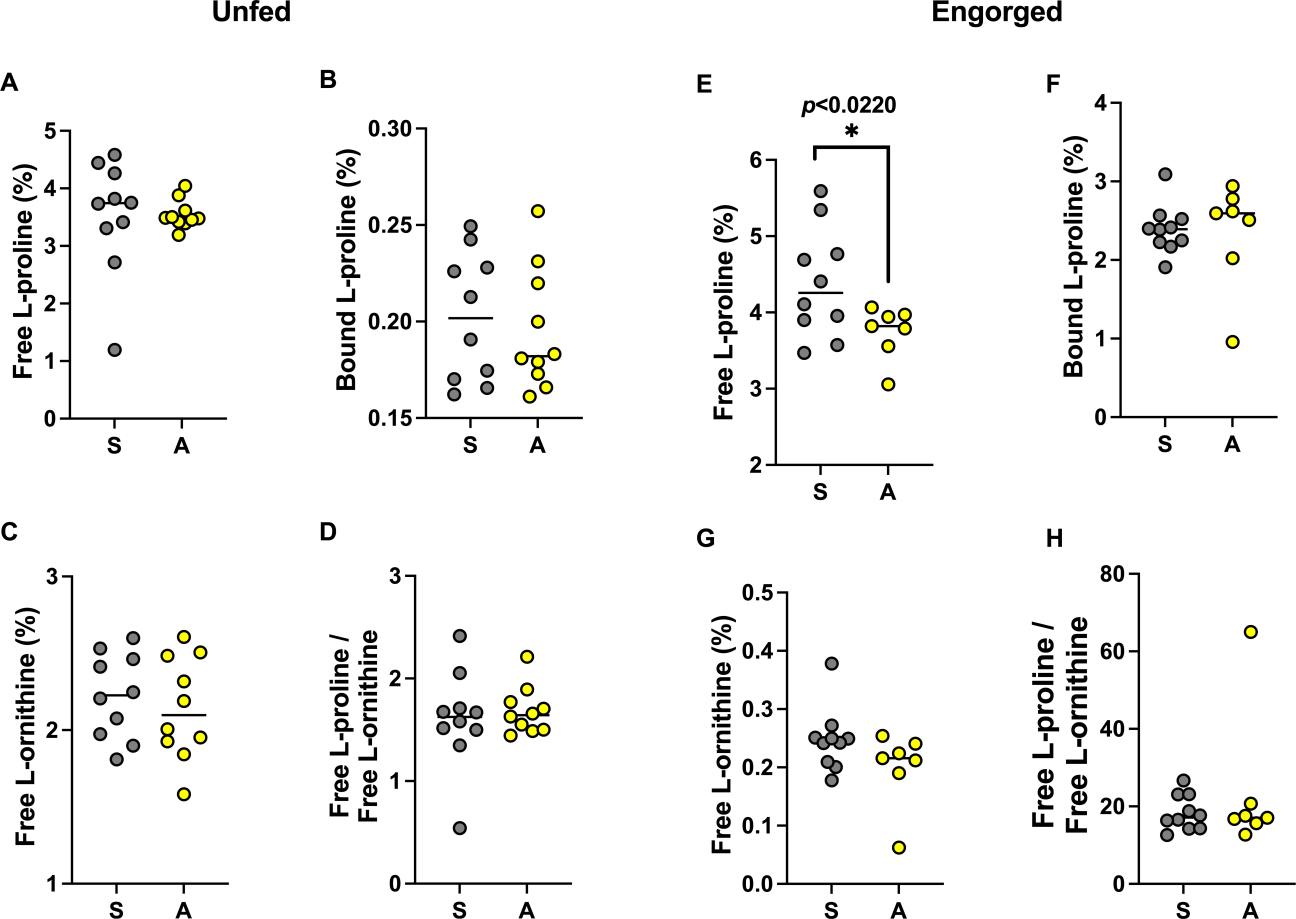
Comparison of normalized (percentage of total amino acids) L-proline and L-ornithine titers and their ratio in saline-treated (S, gray) and antibiotic-treated (A, yellow) unfed ticks (**A–D**) and engorged ticks (**E–H**). Normalized free L-proline (**E**) was significantly higher in saline-treated engorged ticks (*P < 0.0220, *t* = 2.769). *n* = 10 in each parameter tested for unfed ticks and engorged saline-treated ticks, and *n* = 9 in each parameter tested for engorged antibiotic-treated ticks; horizontal line depicts the median.

Data for 34 amino acids (Table S2) were obtained to test our hypothesis; we focus here on L-proline and L-ornithine in whole ticks. Regardless of treatment, unfed adult ticks showed no differences in bound L-proline, free L-proline, free L-ornithine titers, and the ratio between them (Fig. 3A through D). However, the titer of L-proline found in engorged ticks from the saline treatment was significantly higher than in antibiotic-treated ticks, hinting that CLE is involved in excess production of L-proline (Fig. 3E). No other titers or ratios differed significantly between the treatments (Fig. 3F through H).

### CLE involvement in L-proline synthesis

**Fig 4:**
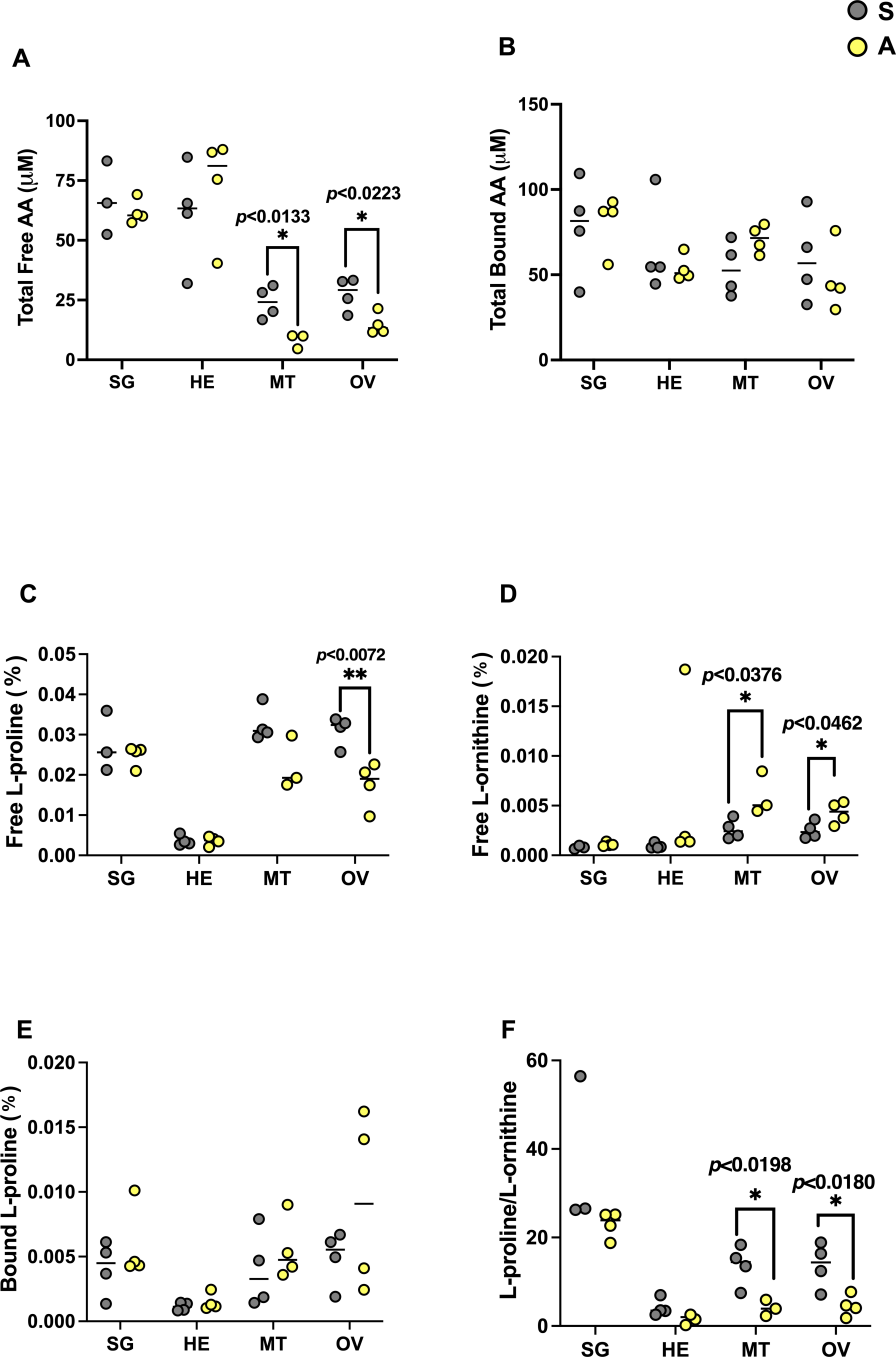
Comparison of normalized (percentage of total amino acids) L-proline and L-ornithine amino acid titers and their ratio in S- (saline, gray) and A- (antibiotic, yellow) treated unfed female tick organs with respect to CLE hosting status. Non-CLE-hosting organs are SG and HE. Naturally CLE-hosting organs are MT and OV. (**A**) Total free amino acids; (**B**) total bound amino acids; (**C**) normalized free proline; (**D**) normalized free ornithine; (**E**) normalized bound proline; (**F**) ratio of free proline/free ornithine. Total free amino acids in CLE- hosting tick organs were significantly higher in saline-treated ticks than in antibiotic-treated ticks (MT *P < 0.0133, *t* = 3.750; OV *P < 0.0223, *t* = 3.058). Free L-proline in OV of symbiotic ticks is significantly higher than in OV of antibiotic-treated ticks (**P < 0.0072, *t* = 3.988). Free L-ornithine in CLE-hosting tick organs was significantly higher in antibiotic-treated ticks (MT *P < 0.0376, *t* = 2.809; OV *P < 0.0462, *t* = 2.506). The ratio of free proline/free ornithine in CLE-hosting tick organs was significantly higher in saline-treated ticks (MT *P < 0.0198, *t* = 3.376; OV *P < 0.0180, *t* = 3.226). Each sample/dot (*n* = 4) contains a pool of four to five organs; horizontal line depicts the median.

To test the hypothesis that CLE synthesizes L-proline (12, 13), we compared the titers of L-proline and L-ornithine (the precursor of L-proline via the ornithine deaminase enzymatic reaction) in organs hosting CLE and in organs that are not known to harbor CLE in *R. sanguineus* in both treatment groups. We first quantified total free and bound amino acids and showed that CLE-hosting organs (Malpighian tubules and ovaries) show higher titers of free amino acids in the saline treatment group (Fig. 4A). There was no difference between the treatment groups in non-hosting organs (hemolymph and salivary glands), and total bound amino acids in all organs did not differ between treatments (Fig. 4B). Free L-proline titer was higher in Malpighian tubules and significantly higher in ovaries of saline-treated ticks than in the same organs from antibiotic-treated ticks (Fig. 4C), and the titers of L-ornithine were significantly lower in CLE-hosting organs from saline-treated ticks than from ticks treated with antibiotics (Fig. 4D). No significant difference was observed for bound proline in the symbiotic organs (Fig. 4E). The ratio of free L-proline to free L-ornithine was significantly higher in CLE-hosting organs of saline-treated ticks than in those of antibiotic-treated ticks (Fig. 4F). This may indicate that L-ornithine is converted to L-proline in the CLE-hosting organs.

### Effect of antibiotics and pre-feeding rescue treatments on feeding duration and weight of adult female ticks

**Fig 5:**
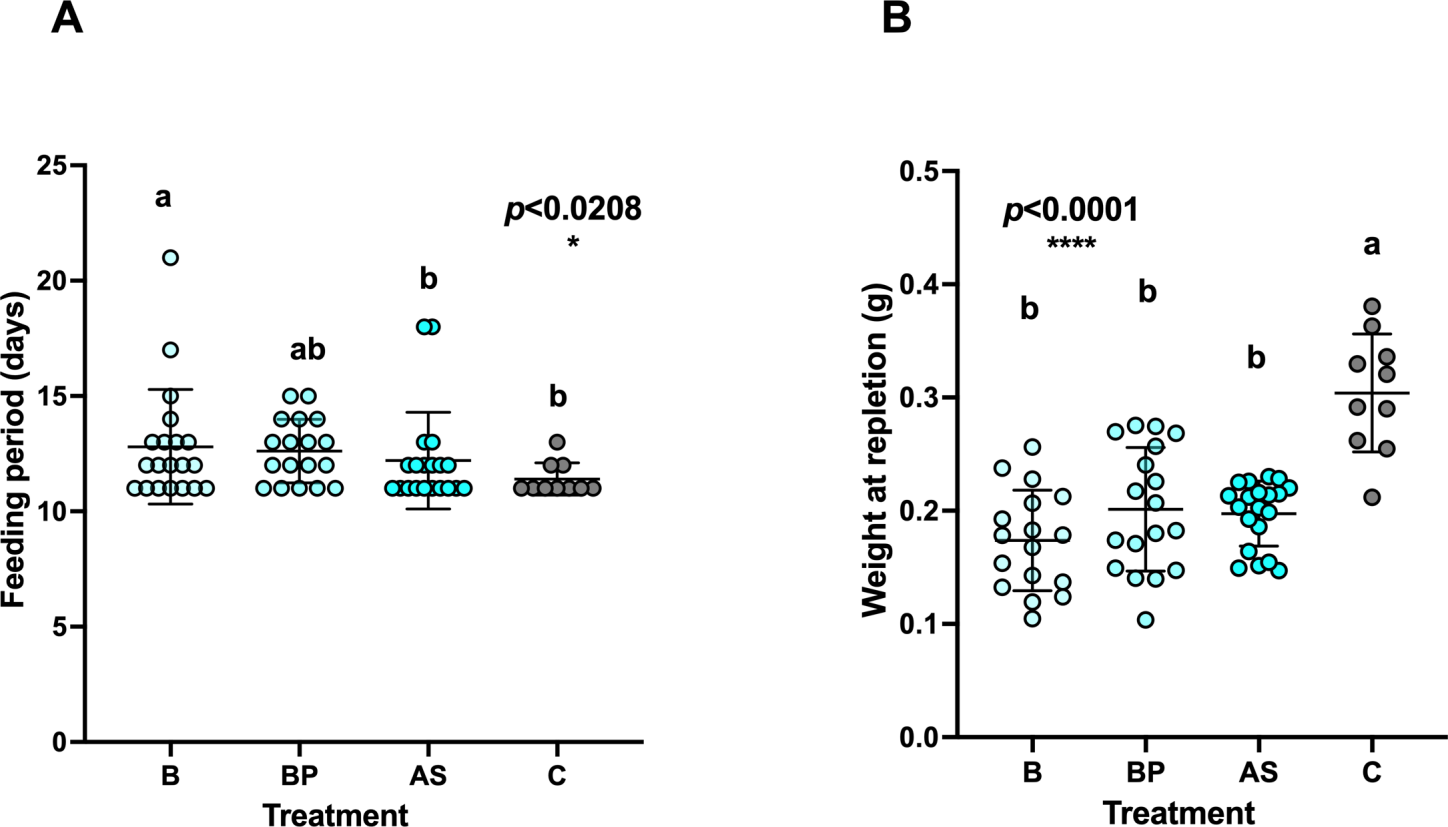
(**A**) Feeding duration of antibiotic-treated ticks followed by rescue treatments and control (B, B vitamins; BP, B-vitamins + L-proline; AS, saline; and C, control) [*H* (3) = 9.759, *P* = 0.0208]. A significant difference was observed among treatments, where B differs from AS and C groups. The control group repleted earlier than the rescue treatment groups. (**B**) Weight of female ticks at repletion following antibiotic and rescue treatments. Control ticks showed significantly increased body mass when compared to the treated ticks (ANOVA, “weight at repletion” *F* = 19.14, DF = 3, *P* < 0.0001). The engorged tick images are presented in Fig. S3.

Adult females from all treatment groups were allowed to mate and feed on rabbit hosts for 3 weeks. The feeding period lasted for 11–21 days until repletion, with a trend of faster molting of the control group, which was statistically different from the B treatment group (Fig. 5A). While the majority of the ticks repleted naturally from the host within 21 days of the onset of the experiment, one female rescued with B and two females rescued with BP failed to replete within 21 days and were not engorged. On average, the non-rescued ticks (injected with saline after antibiotic treatment, termed AS) fed for 12.2 ± 0.42 days, the B-rescued ticks fed for 12.8 ± 0.46 days, and BP-rescued ticks fed for 12.6 ± 0.44 days. Control ticks fed for 11.7 ± 0.43 days, suggesting that the rescue treatment or the microinjections may have a negative influence, which shortens the duration of feeding.

Despite the similar duration of feeding seen among rescue and control groups, we observed that most rescued and non-rescued ticks did not fully engorge, compared with the control group. This was reflected in their weight, where regardless of rescue treatment, CLE- suppressed ticks weighed significantly less than the control ticks (Fig. 5B; 0.17 ± 0.01 g, 0.20 ± 0.01 g, 0.19 ± 0.01 g, 0.304 ± 0.01 g, for the B, BP, S, and C treatments, respectively). Aside from their lower weight, the suppressed CLE ticks showed abnormal features, including wrinkled body surface and darker color due to lack of vitamins (Fig. S3). These results indicate that the rescue treatment was not effective in enabling normal feeding and weight gain of ticks.

### Effect of post-feeding rescue treatments on fecundity and fertility of engorged ticks

**Fig 6:**
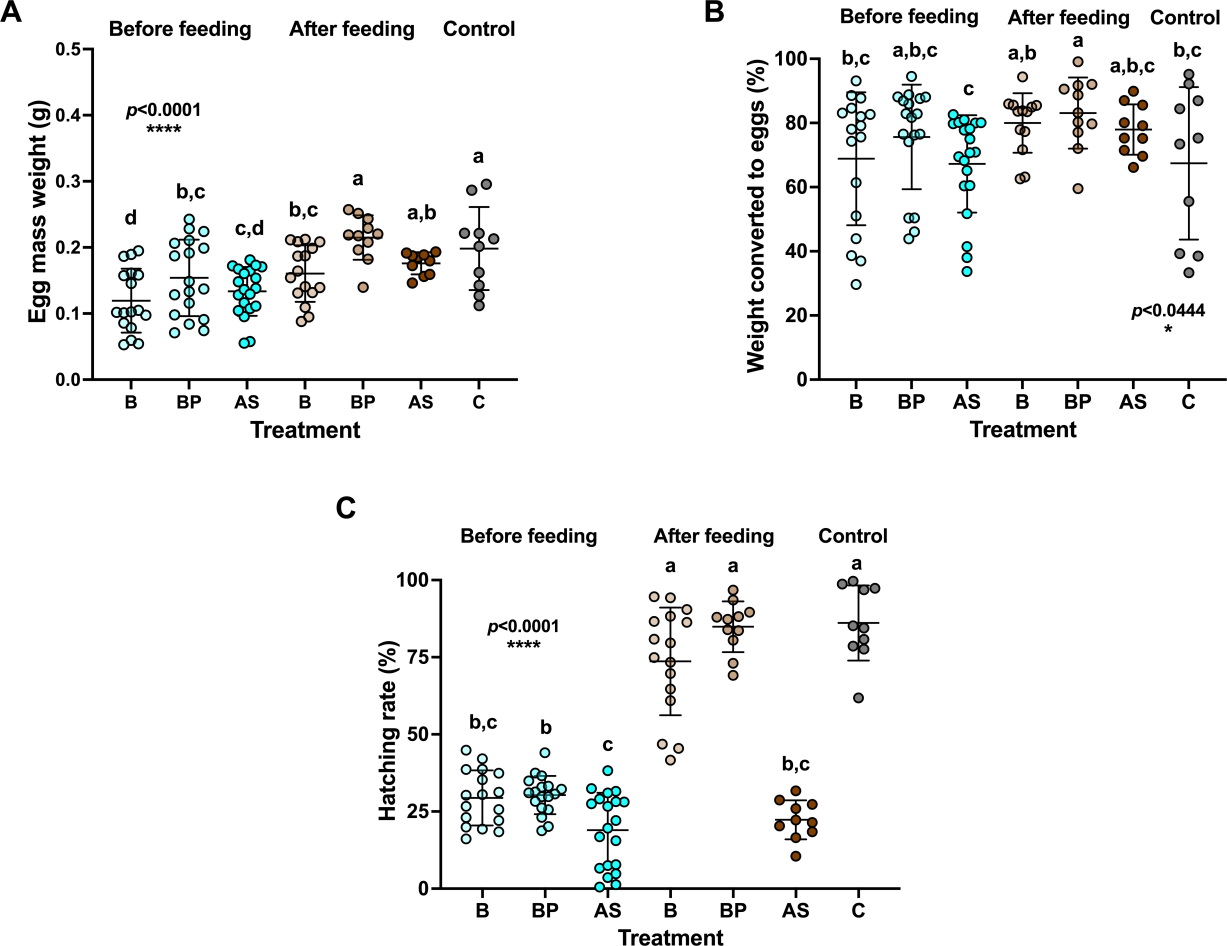
(**A**) Weight of tick egg masses prior to and after rescue and control feeding treatments (B, B vitamins; BP, B vitamins + L-proline; AS, saline; and C, control). The ticks rescued after feeding showed a significant increase in egg mass weight (ANOVA, *F* = 8.16, DF = 6, *P* < 0.0001). (**B**) Proportion of tick weight accounted for by egg production among treatment groups and controls. Ticks rescued after feeding showed a significant increase (Kruskal-Wallis test followed by Wilcoxon; *P* < 0.0444). (**C**) Hatching rate of tick eggs in pre-and post-feeding rescue treatments and the control. Ticks rescued after feeding showed a significantly higher hatching rate (ANOVA, *F* = 100.8, DF = 6, *P* < 0.0001).

Compared to the pre-feeding rescue treatment, the engorged rescue-treated ticks showed increased egg mass weight. The engorged ticks rescued with BP had egg mass weight similar to that of the control ticks (Fig. 6A; BP: 0.21 ± 0.01 g and C: 0.22 ± 0.01 g). The lowest egg mass weight was observed in the pre-feeding rescue treatment (B: 0.11 ± 0.01 g and BP: 0.15 ± 0.01 g). These results suggest that CLE is essential for tick fecundity, probably via B vitamin and L-proline supplementation. To determine egg production efficiency, we divided egg mass weight by the weight of post-oviposition female ticks. The efficiency of egg production varied among treatments, with increase in ticks rescued after feeding (Fig. 6B).

Fecundity was partially rescued by supplementing engorged ticks with B and/or BP; in contrast, fertility, reflected by the hatching rate, was fully rescued by both treatments given to engorged ticks (Fig. 6C). The egg hatching rate of the BP rescued ticks was similar to that of the control ticks (84.8% ± 3.3%; 86.1% ± 3.5%, respectively), followed by B-rescued ticks (73.65% ± 2.7%). As expected, low hatching rates were observed among the B- and BP-treated pre-feeding rescue ticks (29.4% ± 2.6% and 30.3% ± 2.6%, respectively). These findings demonstrate a vital role for B vitamins and L-proline during embryonic development and show that they are probably supplied by CLE.

## DISCUSSION

The supplementation of B vitamins to obligatory blood-feeding arthropods by their dominant symbiotic bacteria is well established (5) but has not been demonstrated experimentally in hard ticks due to technical constraints. It has recently been suggested that additional metabolites, including L-proline and serotonin, may be involved in this symbiosis (13, 20). Here, we demonstrate that L-proline titers are elevated in symbiotic engorged whole ticks and in the organs that host *Coxiella-*like organisms, supporting the involvement of CLE in L-proline production. We also showed that providing supplementary B vitamins to the ixodid tick *Rhipicephalus sanguineus* can rescue the reproductive performance of female ticks deprived of their obligatory CLE. Moreover, the addition of L-proline to the B vitamin supplements rescued fecundity and positively affected fertility.

Hard ticks (Ixodidae), particularly those with short mouthparts (*Gnathostoma*), present difficulties in artificial feeding systems. For this reason, the classic experimental design in which obligatory symbionts are removed from the host, and the effect of supplementing purported symbiont-derived metabolites during feeding is tested, is inapplicable to many Ixodids. Based on our previous findings (6), we instead chose to inject ticks twice with the tested metabolites (B vitamins and L-proline; herein, B and P) in order to mitigate the reduced fitness of CLE-suppressed molted ticks. The first injection was performed prior to attachment and feeding on the animal host in order to evaluate weight gain and subsequent reproductive fitness; the second was done after feeding and repletion to test the rescue of reproductive fitness after feeding. The pre-feeding rescue treatment did not affect feeding performance, and the ticks did not gain weight as in the control. Subsequent fitness measurements were lower than the control. The timing of this treatment imposes limits on injection volume because of the ticks’ small size; the volume of metabolite supplements that we were able to inject could not support the demands of either B or BP supplementation required during *ca*. 20 days of feeding. Alternatively, it is possible that B vitamins are not required for feeding success in *R. sanguineus* and that other CLE-dependent factors are required instead. In support of the first explanation, recent studies on *Ixodes ricinus,* for which artificial feeding is possible, demonstrated that ticks feeding on blood treated with antibiotics performed better when the blood was constantly supplemented with B vitamins during feeding to repletion (21, 22). This was also demonstrated in the soft tick *Ornithodoros moubata* (Argasidea) feeding on blood that was continuously supplemented with B vitamins using an artificial feeding system after the ticks were treated with antibiotics (17).

The second rescue treatment was timed after repletion from the host. Although the tick repletion weight is lower at this point than that of the control, a higher volume of metabolites can be injected, and we can expect better fecundity and fertility than in the non-rescued ticks. This approach did show a rescue effect for tick fecundity, where BP-treated ticks did not differ from the controls, as well as for tick fertility, where the percentage of hatching in the rescue treatments for both B and BP was the same as for the control ticks, with a slightly better performance in the BP treatment. These findings support our hypothesis that CLE is needed at times of extensive cellular activity and high energy demands, during, for example, oogenesis and embryonic development and that their contribution is mediated by the supplementation of B vitamins. The requirement for B vitamins as cofactors in development and reproduction has been demonstrated in various obligatory blood feeder-symbiont systems, among them soft ticks (5). Nevertheless, it should be noted that the rabbit host we worked with is not the main host for these ticks; on a natural host such as dogs, the outcome may have been different.

We have previously shown *in silico* that L-proline is over-produced by CLE and is presumably secreted to the environment (12). We further demonstrated that ornithine cyclodeaminase (OCD), the enzyme that converts L-ornithine to L-proline, is produced in symbiont-hosting organs of ticks (13), and we here support these findings with measurements of L-proline titers. Total free amino acids and the titers of L-proline in symbiotic unfed whole ticks were similar to those found in CLE-reduced ticks. In dissected organs of unfed ticks, however, significantly larger amounts of amino acids and higher titers of L-proline were found only in CLE-hosting organs (Malpighian tubules and ovaries) of symbiotic ticks. Furthermore, the titers of L-ornithine were significantly lower in these organs in symbiotic ticks, indicating that L-proline is additively produced by CLE, presumably via OCD. *putA* and *proC* genes for the biosynthesis of L-proline have been found in the CLE genome (7) and were also detected in its proteome (13), suggesting that L-proline can also be synthesized from glutamate. Other sources can also account for the L-proline supply, including remnants of digested blood meals and tick self-production via genome-encoded enzymes that utilize a pyrroline-5-carboxylate precursor for L-proline synthesis (23). Overall, it can be postulated that L-proline production by CLE is required for CLE maintenance in the hosting organs when the tick is under low metabolic and energetic demands; this is especially true in the cellular environment of Malpighian tubules, the excretory organs, where L-proline may contribute to osmoregulation, as has been seen in other bacteria (24–27). In contrast, higher L-proline titers in symbiotic engorged ticks may account for the L-proline requirement during high physiological demands shown in the rescue experiment. Since we could not detect L-ornithine titer reduction in symbiotic engorged ticks, we cannot conclusively assign the synthesis of L-proline to CLE alone. Unfortunately, dissection of organs from engorged ticks was challenging due to the large amount of blood present, making it difficult to disentangle the source of L-proline in these ticks. Nevertheless, the results support the significance of L-proline synthesis by CLE, which probably contributes to the high energy demand phases in the tick lifecycle. This conclusion may also be supported by the proliferation of three tick cell lines when supplemented with L-proline (28).

Oxidation of proline can serve as an alternative energy source in animals (29), as has been demonstrated in the flight muscles of mosquitoes, tsetse flies, and other insects (30–33). Proline may be an alternative energy source during periods of high metabolic demands for ticks as well; for most of their life cycle, metabolism is low, with high metabolic activity occurring for short periods (34). During embryogenesis, for example, the main energy source is glycogen. Since it is quickly depleted (35), L-proline supplied by CLE may serve as an alternative energy source.

In addition to its role as an energy source, proline may serve various functions in arthropods that can be of relevance during the tick life cycle. It has been suggested, for example, that proline may act as a nitrogen sink in mosquitoes, furthering energy production (36, 37), although in ticks, in contrast, digestion is intracellular (34). In the tsetse fly, the production of proline is symbiont-dependent, and low L-proline levels are associated with reduced fertility (3, 38). Proline may also assist in coping with heat stress associated with heat shock protein production in *Daphnia magna* (39) and proline titer elevation under heat stress in *Zaprionus indianus* (40). Since ticks deal with temporary heat stress during feeding due to exposure to host body temperature (41), it is possible that proline plays a role in temperature regulation during feeding. Interestingly, the cement that ticks secrete and use for attachment to the host (42) contains proline-rich proteins (43) and may also have a role in adjustment to host temperature. These possible functions and the relative contribution of the symbiont to proline titers require specific testing in ticks. Currently, without other evidence, we can only postulate that L-proline serves as an energy source in ticks and that L-proline derived from CLE is an important source of supplementation for improving tick fitness.

## SUPPLEMENTAL MATERIAL

Supplemental figures msphere.00693-23-s0001.docx Figures S1-S3.

Table S1 msphere.00693-23-s0002.xlsx Raw data for the rescue experiments.

Table S2 msphere.00693-23-s0003.xlsx Raw data of biological parameters, metabolites measured, and CLE titers.

Table S3 msphere.00693-23-s0004.xlsx Metabolites of tick organs.

ASM does not own the copyrights to Supplemental Material that may be linked to, or accessed through, an article. The authors have granted ASM a non-exclusive, world-wide license to publish the Supplemental Material files. Please contact the corresponding author directly for reuse.
