# Supplementary material for: Endosymbiont-derived metabolites are essential for tick host reproductive fitness
Source: mSphere. 2024 Jul 2;9(7):e00693-23. doi: 10.1128/msphere.00693-23 (PMC11288044; doi:10.1128/msphere.00693-23)
Supplement: Supplemental figures — Figures S1-S3. [file msphere.00693-23-s0001.docx]

**Supplementary Information**

**Endosymbiont-derived metabolites are essential for its tick host reproductive fitness**

Balasubramanian Cibichakravarthy, Neta Shaked, Einat Kapri, Yuval Gottlieb

**
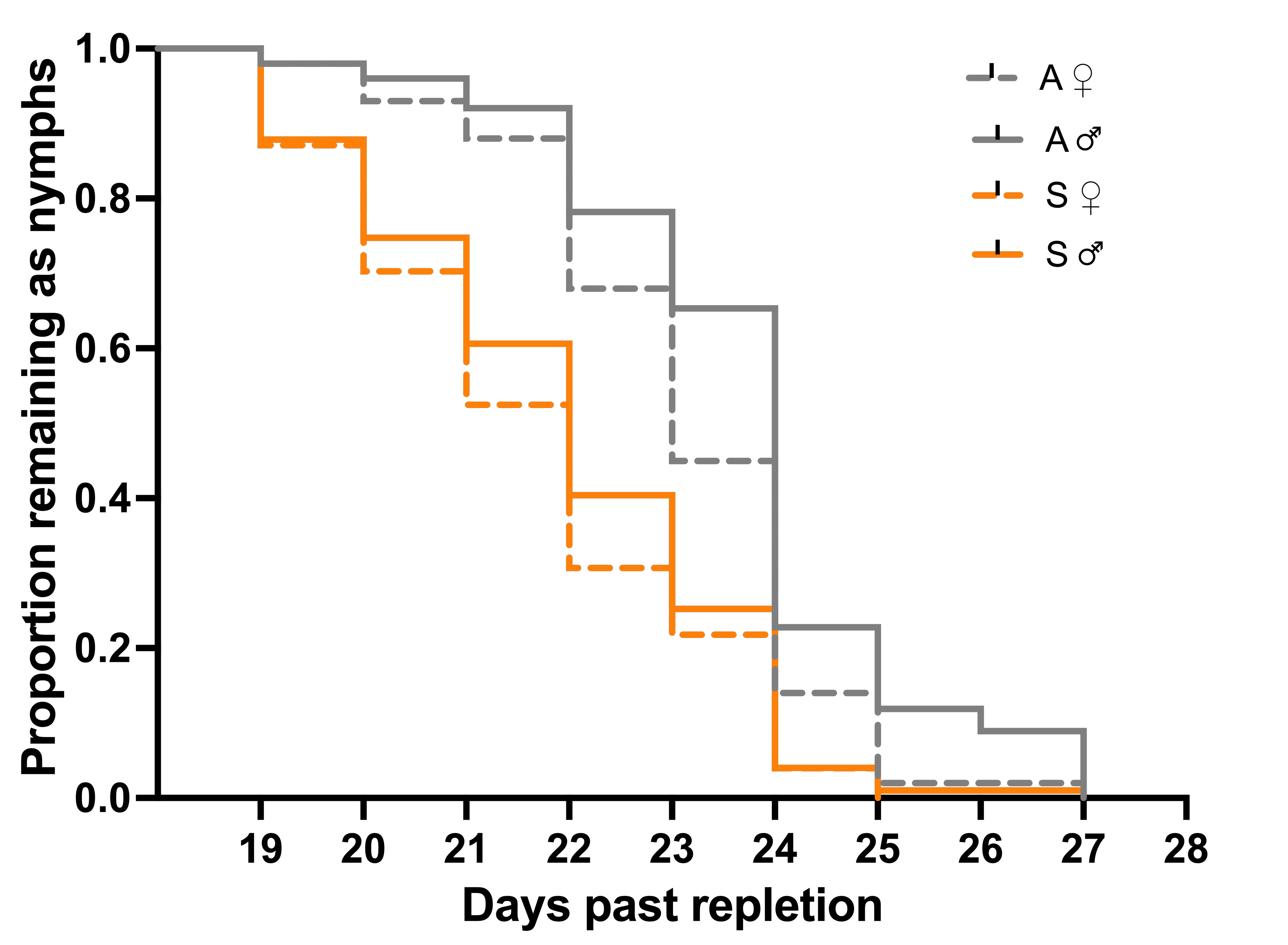
**

Fig. S1. Effect of ofloxacin and saline (control) on the survival of nymphs, n=1020; log-rank test, χ^2^=36.47, df = 3; Cox’s model, **** *p*<0.0001. Line coloration, symbols and patterning signifies the experimental treatment. A, antibiotic treated, S, saline treated, ♀ - female, ♂ - male


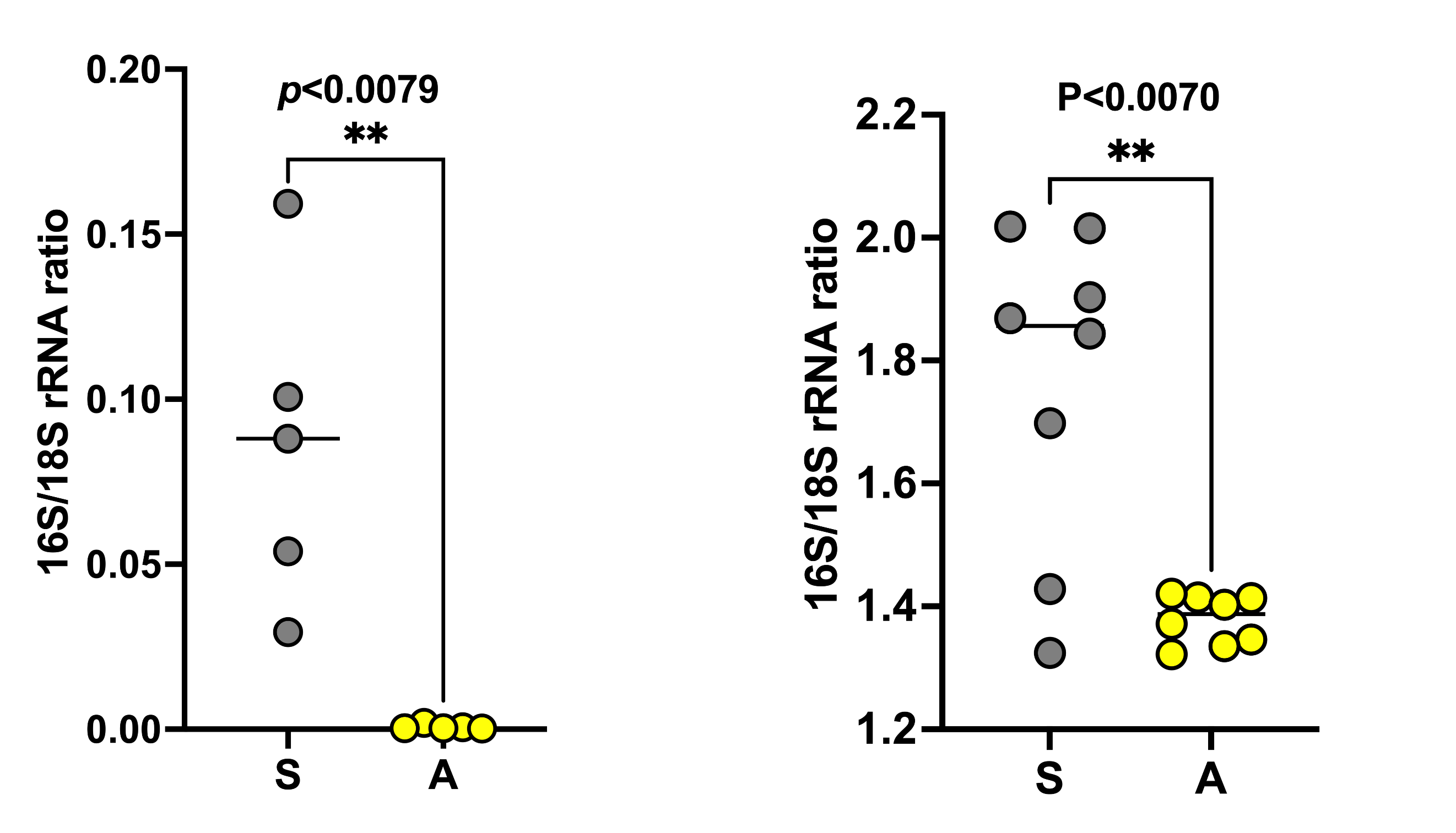


Fig. S2. Relative density of CLE in *R. sanguineus* ticks after ofloxacin treatment in unfed female (A) and in engorged adult female (B) ticks: The changes in CLE density were measured in terms of the number of 16S rRNA gene copies per 18S rRNA gene copies and mean values are shown. ** Indicates significant difference between antibiotic treatment and control at a given dose based on Student t-test (*p*<0.0079), (*p*<0.0070). S- Saline and A- Antibiotic treated.

Fig. S3. The engorged tick images represent the morphological status of ticks. The lack of B vitamins was clearly witnessed by the abnormal small body size and cuticle features including wrinkled surface and darker color. (B- Bvitamins; BP – Bvitamins + l-proline; AS- saline; C- control).
